# The impact of sublethal antimicrobial blue light on lipidomic changes of *Escherichia coli* and *Salmonella* Typhimurium

**DOI:** 10.3389/fcimb.2025.1612638

**Published:** 2025-11-26

**Authors:** Jiaxin Wu, Xuan Lu, Ming Huang, Shuyan Wu, Maomao Zeng, Zhaojun Liu, Keyang Yu, Xiujuan Zhang, Xiaoying Sun, Xiaoyuan Wang, Xiaoqing Hu

**Affiliations:** 1 School of Biotechnology, Jiangnan University, Wuxi, China; 2 School of Modern Engineering and Applied Sciences, Nanjing University, Nanjing, China; 3 AgResearch Ltd., Hopkirk Research Institute, Massey University, Palmerston North, New Zealand

**Keywords:** antimicrobial blue light, lipidomics, *Escherichia coli*, *Salmonella* Typhimurium, unsaturated lipids

## Abstract

Antimicrobial blue light (aBL) is an effective non-thermal disinfection method against foodborne bacterial pathogens. While aBL-induced oxidative stress is known to cause cell membrane damage, its specific effects on bacterial lipid composition remain poorly understood. This study investigated aBL-triggered lipidomic changes in two major foodborne pathogens (Escherichia coli and Salmonella Typhimurium). We identified more than 70 lipid species across 6 classes, with over 50% showing significant variations in profile, intensity, and degree of unsaturation following aBL exposure. Phosphatidylethanolamine (PE) species exhibited the most pronounced alterations. Notably, both strains demonstrated decreased unsaturated lipid intensity alongside increased malondialdehyde levels—a key oxidative stress marker. These findings provide crucial insights into bacterial lipid dynamics under aBL stress and advance our understanding of the molecular mechanisms behind aBL-mediated bacterial inactivation.

## 1 Introduction

Antimicrobial blue light (aBL), with in the spectrum of 400–470 nm, has shown effective antimicrobial properties against a variety of pathogens, including Gram-negative bacteria such as *Escherichia coli, Salmonella enterica, Acinetobacter baumannii, Stenotrophomonas maltophilia, Pseudomonas aeruginosa, Elizabethkingia meningoseptica* and *Helicobacter pylori*, and Gram-positive bacteria such as *Staphylococcus aureus* and *Enterococcus faecium* (Bumah et al., 2015; Dos Anjos et al., 2019; Enwemeka et al., 2008; Halstead et al., 2016; Mussi et al., 2010). The antimicrobial effectiveness of aBL has also led to its application in the preservation of several foods (Josewin et al., 2018; Kim et al., 2017a), including milk (Dos Anjos et al., 2020), eggs, and meat products (Guffey et al., 2016; Kim et al., 2017b; Özyürek et al., 2020). Our previous work showed that 415 nm-aBL inactivated 4 meat-borne pathogens (*E. coli, S. enterica*, *S. aureus* and *Cronobacter sakazakii*) on fresh beef and reduced the bacterial load of *S.* Enteritidis on eggshell surfaces by 5.19 log CFU/mL (Hu et al., 2021a; Luo et al., 2022).

The bactericidal mechanism of aBL is known to involve endogenous photosensitizing chromophores that, when excited by aBL (Dai et al., 2012), generate substantial reactive oxygen species (ROS) (Dai et al., 2012). These ROS subsequently damage critical intracellular biomolecules including DNA, RNA, and proteins (Kim and Yuk, 2017). Our previous studies (Chu et al., 2019; Wu et al., 2018) demonstrated that aBL exposure causes structural damage to cellular surfaces - particularly the cell wall and membrane - in both methicillin-resistant *S. aureus* and *C. sakazakii*, while also significantly reducing unsaturated fatty acids in these strains. Nevertheless, the comprehensive lipidomic alterations in foodborne pathogens under sublethal aBL irradiation remain largely unexplored.

In Gram-negative bacteria, the majority of lipids are embedded within the cell membrane, which were characterized by a diverse array of phospholipids (Zhang and Rock, 2008). The major structural phospholipids, based on their structures (Lin and Weibel, 2016), are mainly classified into phosphatidylethanolamine (PE), phosphatidylglycerol (PG), and cardiolipin (CL). Compared with them, phosphatidylcholine (PC) and phosphatidylinositole (PI) are less prevalent but essential in the lipid synthesis pathway (Sohlenkamp and Geiger, 2016). The lipid profiles in this study were determined by UHPLC-ESI-MS/MS, following methods described previously (Gao et al., 2019; Gidden et al., 2009). Additionally, mass spectrometry methods such as MALDI-TOF/TOF have been applied in lipid analysis for *E. coli* and *B. subtilis*, highlighting their utility in lipidomic studies. In this study, we focus on analyzing changes in phospholipids during aBL irradiation, with quantitative lipid analysis conducted using liquid chromatography-mass spectrometry.

Previously, lipidomic studies has been employed to examine lipid alteration in *E. coli* and *Staphylococcus warneri* during photodynamic therapy (PDT) based on Tri-Py^+^-Me-PF (Alves et al., 2013a, Alves et al., 2013b). These studies identified membrane lipids as key molecular targets of PDT. Alves et al. reported that PGs, a dominant lipid in *S. warneri*, showed an overall increase in the abundance.

Unlike PDT, aBL achieves antibacterial effects without the need for exogeneous photosensitizers. This study aims to investigate the impact of aBL on the lipidome in two clinically relevant pathogens (*E. coli* and *Salmonella* Typhimurium) following sublethal aBL treatment. By elucidating how sublethal aBL exposure modifies bacterial lipid profiles, we aim to uncover the relationship between light-induced oxidative stress and bacterial susceptibility. These insights could inform the development of optimized light-based disinfection strategies for enhanced pathogen control in clinical and industrial settings.

## 2 Materials and methods

### 2.1 Strains and culture condition

Both *E. coli* W3110 and *S.* Typhimurium SL1344 were purchased from ATCC. All of the stains were cultivated at 37°C on Luria-Bertani agar plates containing 10 g/L tryptone 5 g/L yeast extract, 10 g/L NaCl and 15 g/L agar. Subsequently, a single colony was selected and inoculated into 5 mL of LB broth, then incubated at 37°C with shaking at 200 rpm. Cells were harvested once the optical density at 600 nm (OD_600_) reached the desired level for further investigation.

### 2.2 Sub-lethal irradiation by aBL

The procedure of aBL irradiation was carried out using a method previously reported (Wu et al., 2018). The aBL irradiation was performed using a 415 nm LED array (Omnilux clear-UTM, Photo Therapeutics, Inc.) with a peak wavelength of 415 nm and a bandwidth of 20 nm. The LED array was positioned at a fixed height to ensure an irradiation intensity of 16.7 mW/cm². The cell suspension with OD_600_ at 2.0 was prepared for aBL treatment testing. Five mL of cell suspension was transferred to a well of a 6-well clear flat-bottom plate (36 mm diameter, 16.5 mm height) and placed on a magnetic stirrer at 30 rpm with gentle stirring. The bacterial suspensions were illuminated with aBL, and the survival rates were measured (Lipovsky et al., 2009; Wu et al., 2018). Aliquots of 20 µL were taken from the aBL-treated cell suspensions at the irradiation doses of 0, 10, 20, 30, 60, 90, and 120 J/cm^2^ respectively. Visible cell counts of the aBL-treated bacterial population were measured and the bacterial cell reduction was calculated by comparison with untreated control group. Cells exposed to the sub-lethal aBL doses of 15 J/cm^2^ and 30 J/cm^2^ were harvested separately to lipid extraction, as detailed in the procedure below.

### 2.3 Extraction of lipids

Lipid extraction from *S.* Typhimurium and *E. coli* cells following aBL treatment was performed using a modified protocol based on the method of (Folch et al., 1957). Thirty mL of cell suspensions were centrifuged at 4,000 × g for 10 min at 4 °C. The cell pellets were washed thrice with ultrapure water, with each wash followed by 5-min of vibration. After washing, the bacterial cells were subjected to three freeze-thaw cycles by alternately immersing them in liquid nitrogen for 1 min and thawing on ice for 3 min to promote lysis. The lysed cells were resuspended in 6 mL of ultrapure water and gently vortexed for 30 s. For lipid extraction, 900 μL of pre-cooled chloroform/methanol (2:1, v/v) was added to the suspension. The mixture was vortexed for 30 s and centrifuged at 4,000 × g for 10 min at 4 °C. The lower organic phase was carefully collected. A second round of extraction was performed using the same solvent system, and both organic phases were combined. The combined extracts were dried under a gentle stream of nitrogen gas. The resulting lipid residues were reconstituted in a mixture of acetonitrile/isopropanol and chloroform/methanol (1:1, v/v), and stored at –20°C until LC-MS analysis.

### 2.4 Isolation and quantitative analysis of lipids by liquid chromatography–mass spectrometry

The Lipid extracts were separated using a UHPLC system (Ultimate 3000, Thermo Scientific, Bremen, Germany) coupled to a ThermoFisher Q Exactive™ Plus Hybrid Quadrupole-Orbitrap™ mass spectrometer. Chromatographic separation was performed using two mobile phases: mobile phase A, composed of acetonitrile and water (60:40, v/v) with 10 mM ammonium acetate, and mobile phase B, composed of isopropanol and acetonitrile (90:10, v/v) with 10 mM ammonium acetate. The sample was injected into a Waters CSH C18 column (2.1 mm × 100 mm, 1.7 μm) and separated using a linear gradient, transitioning from 60% mobile phase A to 95% mobile phase B over 20 minutes, followed by re-equilibration to initial conditions. The flow rate was maintained at 0.25 mL/min. The MS analysis was performed in the negative ion mode with an electrospray voltage of 2.8 kV, a capillary temperature of 350°C, and a sheath gas flow rate of 35 units. MS and MS/MS data acquisition were acquired over an m/z range of 80-1200 (Schwalbe-Herrmann et al., 2010).

### 2.5 Lipid identification and data analysis

**Figure 1:**
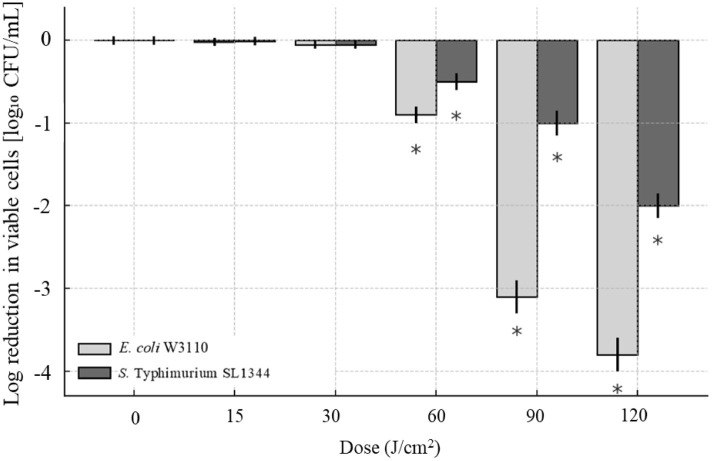
Intensity of lipids changes under different aBL irradiation doses in *E. coli* W3110 **(A)** Overall lipid intensity variation under 0, 15, and 30 J/cm² aBL. There existed significant differences between different samples (*p* < 0.05). “a” meant significant differences between samples under 0 and 15 J/cm², “b” meant that under 0 and 30 J/cm², and “c” meant that under 15 and 30 J/cm²; **(B, C)** Representative lipids exhibiting discontinuous changes in the intensity under different aBL doses.

Lipid species were identified using LipidSearch software based on retention time, accurate mass, and MS/MS spectra. Specific ion forms were used for lipid identification, including [M-2H]^2-^ for CL and [M-H]^-^ for PE, PG, and PS. MS/MS spectra further characterized lipids, particularly for those containing two fatty acid chains. For example, PE(17:0-18:0) was identified by characteristic m/z signals corresponding to its head group and fatty acid carboxylate ions. Normalized data sets were analyzed using MetaboAnalyst 5.0. Partial least squares discriminant analysis (PLS-DA) revealed clear clustering between aBL-treated and control groups, differentiating lipidomic profiles under varying aBL irradiation conditions. All experiments were conducted in triplicate, and results were expressed as mean ± standard deviation (SD). Statistical analyses were performed using Origin with one-way analysis of variance (ANOVA), followed by appropriate *post hoc* testing to evaluate differences between treatment groups. A *p*-value less than 0.05 was considered statistically significant. In **Figures 1** and **5**, lowercase letters above the bars indicate statistically significant differences between specific time points:

- “a” indicates a significant difference between 15 min and 0 min (*p* < 0.05);
- “b” indicates a significant difference between 30 min and 0 min (*p* < 0.05);
- “c” indicates a significant difference between 30 min and 15 min (*p* < 0.05).
- These annotations help to highlight the temporal progression of the observed effects.

### 2.6 Quantification of lipid peroxidation

The procedure of malondialdehyde (MDA) exact and analysis was identical to that previously reported by our group (Wu et al., 2018). The absorbance of the supernatant at 532 nm was measured and MDA content was calculated using a standard curve. The detailed procedure followed the manufacturer’s protocol (Kit #S0103, Beyotime, China). The intracellular ROS was detected based on our previous report (Wu et al., 2018).

### 2.7 Determination of the outer membrane permeability and potassium leakage

OM permeability was assessed using the N-phenyl-α-naphthylamine (NPN) uptake method (Qiao et al., 2021). Following aBL irradiation at different doses (0, 15, and 30 J/cm^2^), the cells were resuspended in 1.92 mL of phosphate buffer solution (OD_600_ = 0.5). Subsequently, 80 μL of NPN solution (1 mM) was added to the cell suspension, and the fluorescence intensity was immediately measured using a spectrofluorometer (Hitachi, Tokyo, Japan). The excitation wavelength was set at 350/10 nm, and the emission wavelength was set at 420/10 nm. Potassium leakage was determined by measuring extracellular K^+^ concentration in the supernatant using a flame photometer after centrifugation at 12,000 × g for 5 min (Wu et al., 2018).

## 3 Results and discussions

### 3.1 Bacterial inactivation of *E. coli* and *S.* Typhimurium upon aBL

**Figure 2:**
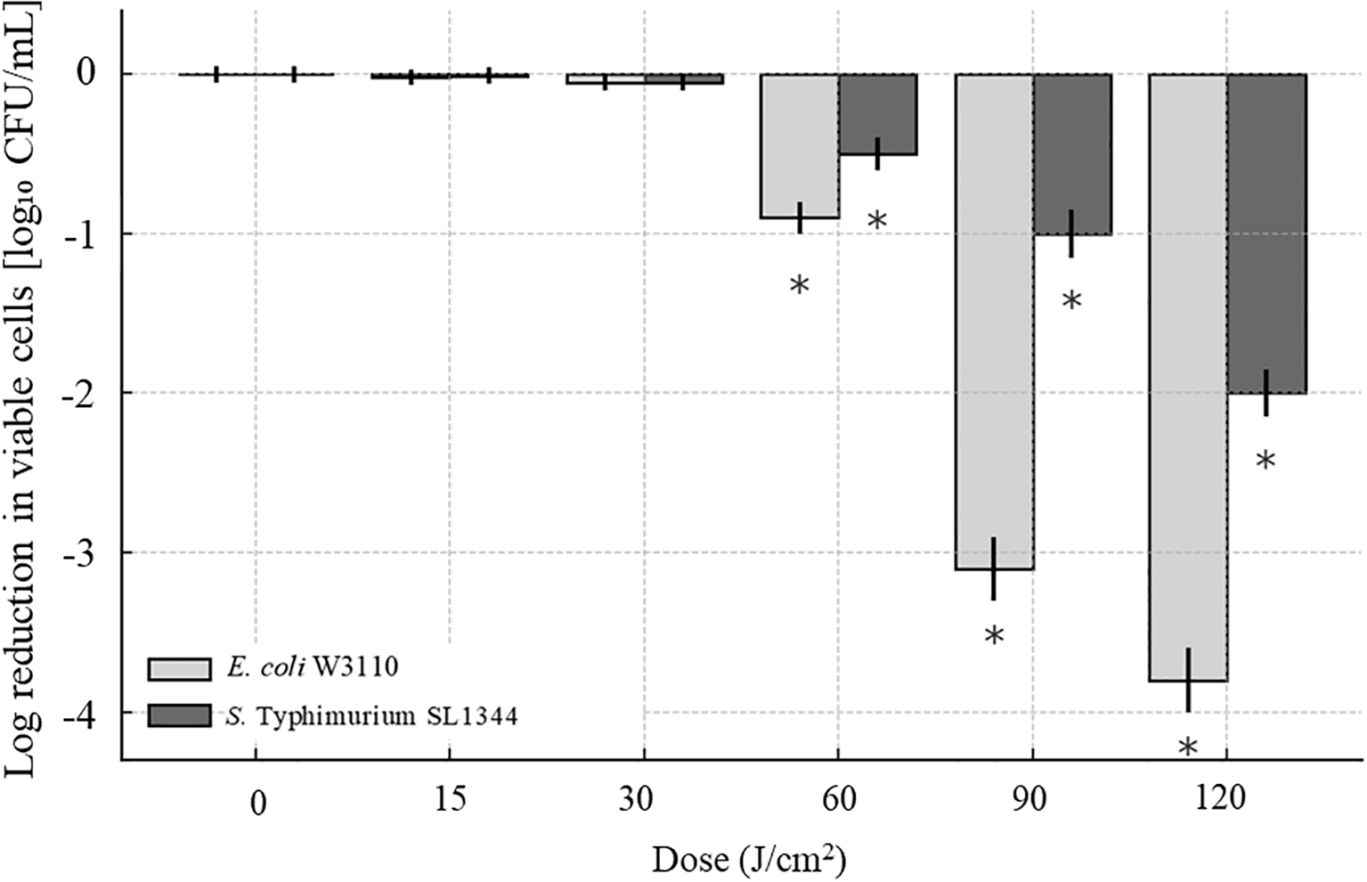
Inactivation of BL against *E. coli* W3110 *and S.* Typhimurium SL1344. Bacterial cultures were exposed to increasing doses of aBL (0–120 J/cm²). The reduction in viable cells was quantified by plating serial dilutions and calculating the log_10_ reduction in colony-forming units (CFU/mL). Data were shown as mean ± SD from three independent experiments. Statistical significance was evaluated using two-way ANOVA, and representative trends were displayed without individual *p*-value markers for clarity.

The aBL exhibited efficient bactericidal effect against *E. coli* W3110 and *S.* Typhimurium SL1344, while two strains exhibited differential sensitivity to aBL in a dose-dependent manner. As shown in **Figure 2**, when aBL dose exceeded 30 J/cm^2^, the population of *E. coli* W3110 began to decrease, and when irradiation was extended to 120 J/cm^2^, it was further reduced by 3.76 log CFU/mL. Previous studies have shown that aBL at various wavelengths produced similar bactericidal effects against *E. coli* isolates. For examples, 410-nm aBL irradiation at 412.50 J/cm^2^ dose killed *E. coli* ST10 beyond 3.7 logCFU and *E. coli* ST648 at 2.8 logCFU, respectively (Dos Anjos et al., 2019). Furthermore, *E. coli* ST131 and O127:H7 became non-culturable following aBL exposure (Dos Anjos et al., 2019). Another study reported that 455-nm aBL illumination at 120 J/cm^2^ reduced 1.5-2.5 log CFU/mL of *E. coli* DH5α and 1 log CFU/mL of *E. coli* MG1655 (Abana et al., 2017). In the present study, 415-nm aBL illumination at 120 J/cm^2^ achieved comparable inactivation efficiency. These variations of bactericidal efficiency of aBL were likely due to differences in strain-specific sensitivity, initial bacterial concentration, culture conditions, and light exposure parameters such as wavelength and irradiance uniformity. In our previous report on *S.* Typhimurium SL1344 (Hu et al., 2021a), even a single mutation of lipopolysaccharide located at OM significantly changed its photosensitivity, thus for these *E. coli* strains mentioned above, substantial differences in genetic background and OM properties plausibly account for the observed discrepancies across studies.

The inactivation of *S.* Typhimurium SL1344 was observed when aBL dose exceeded 60 J/cm^2^ (**Figure 2**). Viable cell counts decreased by 1.03 log CFU/mL after 90 J/cm^2^ and by 2.02 after 120 J/cm^2^. In contrast, our previous study reported 383 J/cm^2^ aBL inactivated SL1344 by 4 log CFU/mL (Hu et al., 2021a). Similarly, other studies reported that 405 nm aBL reduced *S.* enterica serovar Enteritidis by 1.4 log CFU/mL after 739.6 J/cm² exposure (Endarko et al., 2012), and reduced *S.* Enteritidis and *S.* Saintpaul by 2.0 log CFU/mL and 1.0 log CFU/mL, respectively, after 288 J/cm^2^ treatment (Kim and Yuk, 2017).

In summary, our study demonstrated the bactericidal efficacy of 415-nm aBL against both *E. coli* and *S.* Typhimurium, while revealing species-specific susceptibility to aBL treatment. At the maximum aBL dose at 120 J/cm², viable cell counts decreased by 3.76 log CFU/mL for *E. coli* W3110 and 2.02 log CFU/mL for *S.* Typhimurium SL1344. Notably, aBL could be generated using simple LED-based devices without requiring exogenous photosensitizers, and exert bactericidal effect against a broad spectrum of foodborne pathogens. These findings supported aBL as a promising non-thermal disinfection strategy for foodborne pathogens.

### 3.2 Influence of aBL on lipidomics of *E. coli*

#### 3.2.1 Lipid identification and profiling

**Figure 3:**
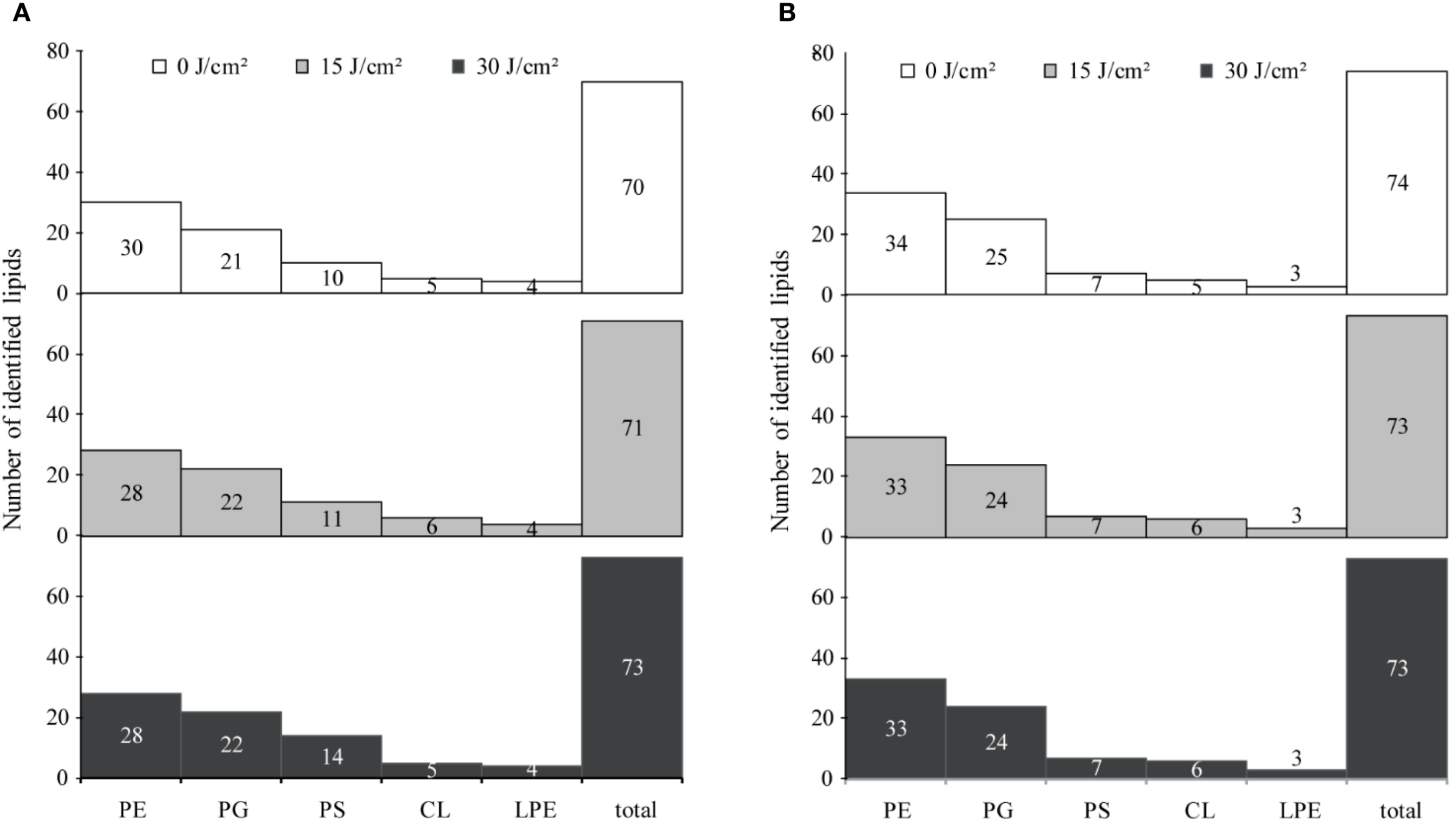
Distribution and number of identified lipid species under different doses of antimicrobial blue light (aBL). **(A)***E. coli* W3110; **(B)***S.* Typhimurium SL1344. The total number of identified lipid species and the distribution of lipid variations were assessed in each strain after treatment with 0, 15, or 30 J/cm^2^ aBL. Changes reflect differences in membrane lipid composition induced by sublethal aBL exposure.

The UHPLC system, equipped with a C18 column, effectively separated lipids from pooled lipid extracts of *E. coli* W3110 cells treated with 0, 15, and 30 J/cm^2^ aBL doses, and the PLS-DA was analysis was showed in **Supplementary Figure S1**. In the untreated *E. coli* cells (the control group), 70 lipid species belonging to 5 lipid classes were identified, including 30 PE, 22 PG, 6 CL, 4 LPE, and 14 PS species. MS chromatograms of these lipids were demonstrated in **Supplementary Figure S2** and **Supplementary Figure S3**, and all lipids information were listed in **Supplementary Table S1**. These lipids comprised of 55 unsaturated lipids and 15 saturated lipids, with the detailed information summarized in **Supplementary Table S2**. In the group treated with 15 J/cm² aBL, a total of 71 lipid species were detected. Compared to the control group, four lipid species were absent, including two PSs [PS(16:0-8:0), PS(16:0-19:2)] and two PEs [PE(16:1-14:1), PE(17:1-12:0)]. Five lipids were uniquely detected in the treated group, including three PSs [PS(16:0-17:1), PS(16:1-17:2), PS(17:1-17:2)], PG(18:1-18:1), and CL(18:1/16:1/16:0/18:1) (**Figure 3A**). In the treated cell group irradiated by 30 J/cm^2^, 73 lipid species were detected (**Figure 3A**). This light dose induced the emergence of 3 PSs [PS(16:0-19:2), PS(19:2-14:1), PS(18:1-19:2)], while CL(18:1/16:1/16:0/18:1) became undetectable, these changes were not observed in the control group. The number of PEs and PGs remained consistent at 28 and 22, respectively.

In *E. coli*, cell membrane lipids, including both the outer and inner membrane, constitute approximately 95% of total lipids (Silhavy et al., 2010). The biosynthesis of these lipids follows conserved pathways shared between eukaryotes and prokaryotes. For instance, PA, synthesized from glycerol-3-P, serves as precursor of various lipid forms. Through conversion to CDP-DAG, PA gives rise to both the zwitterionic lipid PE and the anionic lipids PG and CL (Dowhan, 2013). Notably, LPE can be produced either during PE synthesis or through the breakdown of bacterial membranes (D’Arrigo and Servi, 2010).

Considering the well-established influence of lipid composition on membrane integrity and aBL sensitivity (Chu et al., 2019; Hu et al., 2021a, Hu et al., 2021b), significant diversity and dynamic changes in global lipids under aBL irradiation were discovered in the subsequent sections.

#### 3.2.2 Variations in lipid intensity during aBL irradiation

Although only minor changes in lipid species composition were observed following aBL exposure, significant shifts in lipid intensity occurred throughout the irradiation process. These changes in the abundance of lipids are summarized in **Tables 1** and **2**.

**Table 1:** The continuous changes in specific lipid species of *E. coli* W3110 during aBL treatment.

Dynamic trends of lipid species under different conditions	Lipidion	Adduct	Formula	M/z
Lipid species showing continuous increase	CL (16:0-16:1-16:0-16:1)	[M − 2H]^2-^	C_73_ H_136_ O_17_ P_2_	673.463189
	CL (18:1-16:1-16:0-18:1)	[M − 2H]^2-^	C_77_ H_142_ O_17_ P_2_	700.486664
	PE (16:0-12:0)	[M − H]^-^	C_33_ H_65_ O_8_ N_1_ P_1_	634.445331
	PE (16:0-14:0)	[M − H]^-^	C_35_ H_69_ O_8_ N_1_ P_1_	660.476631
	PE (16:0-17:0)	[M − H]^-^	C_38_ H_75_ O_8_ N_1_ P_1_	704.523581
	PE (16:0-18:0)	[M − H]^-^	C_39_ H_77_ O_8_ N_1_ P_1_	718.523581
	PE (19:1-16:0)	[M − H]^-^	C_40_ H_77_ O_8_ N_1_ P_1_	730.539231
	PE (18:0-18:1)	[M − H]^-^	C_41_ H_79_ O_8_ N_1_ P_1_	744.554881
	PG (16:0-12:0)	[M − H]^-^	C_34_ H_66_ O_10_ N_0_ P_1_	665.439912
	PG (16:0-14:0)	[M − H]^-^	C_36_ H_70_ O_10_ N_0_ P_1_	693.471212
	PG (16:0-14:1)	[M − H]^-^	C_36_ H_68_ O_10_ N_0_ P_1_	691.455562
	PG (15:0-16:0)	[M − H]^-^	C_37_ H_72_ O_10_ N_0_ P_1_	707.486862
	PG (17:1-14:0)	[M − H]^-^	C_37_ H_70_ O_10_ N_0_ P_1_	705.471212
	PG (17:1-16:0)	[M − H]^-^	C_39_ H_74_ O_10_ N_0_ P_1_	733.502512
	PG (16:0-18:2)	[M − H]^-^	C_40_ H_74_ O_10_ N_0_ P_1_	745.502512
	PG (18:0-18:1)	[M − H]^-^	C_42_ H_80_ O_10_ N_0_ P_1_	775.549462
	PG (18:1-18:1)	[M − H]^-^	C_42_ H_78_ O_10_ N_0_ P_1_	773.533812
	PS (16:1-19:2)	[M − H]^-^	C_41_ H_73_ O_10_ N_1_ P_1_	770.497761
	PS (17:1-19:2)	[M − H]^-^	C_42_ H_75_ O_10_ N_1_ P_1_	784.513411
	PS (18:1-21:3)	[M − H]^-^	C_45_ H_79_ O_10_ N_1_ P_1_	824.544711
Lipid species showing continuous decrease	CL (18:1-16:1-16:0-16:1)	[M − 2H]^2-^	C_75_ H_138_ O_17_ P_2_	686.471014
	CL (17:1-18:1-16:0-16:0)	[M − 2H]^2-^	C_76_ H_142_ O_17_ P_2_	694.486664
	PE (17:1-18:1)	[M − H]^-^	C_40_ H_75_ O_8_ N_1_ P_1_	728.523581
	PE (12:0-14:0)	[M − H]^-^	C_31_ H_61_ O_8_ N_1_ P_1_	606.414031
	PE (18:1-12:0)	[M − H]^-^	C_35_ H_67_ O_8_ N_1_ P_1_	660.460981
	PE (18:1-14:0)	[M − H]^-^	C_37_ H_71_ O_8_ N_1_ P_1_	688.492281
	PE (16:1-16:1)	[M − H]^-^	C_37_ H_69_ O_8_ N_1_ P_1_	686.476631
	PE (17:1-16:0)	[M − H]^-^	C_38_ H_73_ O_8_ N_1_ P_1_	702.507931
	PE (17:1-16:1)	[M − H]^-^	C_38_ H_71_ O_8_ N_1_ P_1_	700.492281
	PE (17:1-18:1)	[M − H]^-^	C_40_ H_75_ O_8_ N_1_ P_1_	728.523581
	PE (18:1-16:1)	[M − H]^-^	C_39_ H_77_ O_8_ N_1_ P_1_	714.539231
	PE (16:0-18:1)	[M − H]^-^	C_39_ H_75_ O_8_ N_1_ P_1_	716.523581
	PE (17:1-17:1)	[M − H]^-^	C_39_ H_73_ O_8_ N_1_ P_1_	714.507931
	PE (17:1-19:1)	[M − H]^-^	C_41_ H_77_ O_8_ N_1_ P_1_	742.539231
	PG (17:1-16:0)	[M − H]^-^	C_39_ H_74_ O_10_ N_0_ P_1_	733.502512
	PG (16:0-18:1)	[M − H]^-^	C_40_ H_76_ O_10_ N_0_ P_1_	747.518162
	PG (19:1-16:0)	[M − H]^-^	C_41_ H_78_ O_10_ N_0_ P_1_	761.533812
	PS (19:2-14:0)	[M − H]^-^	C_39_ H_71_ O_10_ N_1_ P_1_	744.482111

**Table 2A:** Two distinct patterns of specific lipid species in *E. coli* W3110 upon aBL treatment: **(A)** initial decrease followed by increase, and **(B)** initial increase followed by decrease.

Lipidion	Ratio of lipid intensity		
	0 j/cm^2^	15 j/cm^2^	30 j/cm^2^
PE(15:0-14:0)	1.000	0.722 ± 0.048^a^	1.036 ± 0.087
PG(16:1-16:1)	1.000	0.832 ± 0.187	1.169 ± 0.075^bc^
PG(16:1-18:1)	1.000	0.969 ± 0.021	1.006 ± 0.056
PG(17:1-18:1)	1.000	0.772 ± 0.012^a^	1.011 ± 0.049
PG(18:0-16:0)	1.000	0.954 ± 0.192	1.046 ± 0.072
LPE(16:1)	1.000	0.876 ± 0.032^a^	0.983 ± 0.120
LPE(17:1)	1.000	0.941 ± 0.033	0.958 ± 0.101
LPE(18:1)	1.000	0.920 ± 0.063	1.029 ± 0.070

“a” meant significant difference between samples under 0 and 15 J/cm² (*p* < 0.05); “b” meant significant difference between samples under 15 and 30 J/cm² (*p* < 0.05), “c” meant significant difference between samples 0 and 30 J/cm².

First, over half of the total lipid species exhibited consistent changes of intensity (**Table 1**). Unless otherwise specified, the terms “increase” or “decrease” referred to changes relative to the immediately preceding sample, for example, samples under 15 J vs. 0 J, and 30 J vs. 15 J. During aBL irradiation, firstly, 20 lipid species showed continuous increase in the intensity, including 6 PEs, 9 PGs, and 3 PSs. Especially, CL(18:1-16:1-16:0-18:1) and PG(18:1-18:1) showed significant increase; PE(16:0-18:0) and PG(17:1-16:0) displayed a 2-fold rise (**Figure 1A**). The majority of these 20 lipid species were unsaturated, with 6 mono-unsaturated species and 7 poly-unsaturated species. Secondly, 18 lipid species showed a continuous decrease in intensity, including 12 PEs, 3 PGs, and 1 PS. Except PE(12:0-14:0), the remaining 17 lipids were unsaturated, including 7 monounsaturated species and 10 polyunsaturated species. Notably, PE(16:0-18:1), PG(16:0-18:1), and PE(17:1-17:1) showed the greatest reductions beyond 96.0% (**Figure 1A**). Unsaturated fatty acyl chains are known to be susceptible to ROS-induced peroxidation (Yang and Stockwell, 2016). Upon aBL treatment, lipid peroxidation preferentially targets these unsaturated chains, leading to a significant decrease in their abundance. Similar patterns were observed in the photodynamic inactivation studies reported previously, where PE and PG species containing unsaturated chains were preferentially oxidized (Alves et al., 2013a; Chu et al., 2019). Notably, the lipids across all three biological replicates (**Supplementary Table S1**) showed the similar change trends. For most lipid species showing significant upregulation or downregulation, the direction of change remained consistent, and the variation across replicates was minimal (coefficient of variation < 15%). Statistical analysis indicated significant differences between irradiated sample and the previous one (**Tables 1**, **2**).

Besides, other lipids did not show a continuous increase or decrease trend. On the one hand, as shown in **Tables 2A** and **2B**, there existed 8 lipid species initially decreased in the intensity at 15 J/cm² but subsequently increased at 30 J/cm², which was particularly evident in PE(15:0-14:0) and PG(16:1-16:1). On the other hand, there were other 11 lipid species initially increased while late declined in the level. The most significant example was PE(15:0-16:0), which exhibited the doubled level at 15 J/cm^2^ irradiation while 16.7% lower level after 30 J/cm^2^ of aBL irradiation. Similar trends were also observed for PE(18:1-18:1), PE(19:1-18:1), and PS(16:0-19:2) (**Figure 1C**). The change of these lipid species mentioned above reflected the high variability of global lipids, a major contributor to cell surface damage during aBL illumination. PE lipids exhibited the most pronounced alterations among all classes. This could be attributed to the high abundance of PE in bacterial membranes and its structural propensity to form non-lamellar phases, making it more reactive and susceptible to peroxidative damage under ROS stress (Mileykovskaya and Dowhan, 2009). The reduction of specific PE species may reflect both oxidative lipid damage and active membrane turnover during stress response. Additionally, CL(16:1-16:1-16:0-16:1) became undetectable at 30 J/cm^2^ (**Figure 1A**). These variations likely reflect cell membrane damage, suggesting further investigation into MDA accumulation and OM permeability. Another possible mechanism for lipid alteration may involve cells responding to oxidative stress. Although this response ultimately failed to avert cell membrane rupture and cell death, such changes were manifested by lipids alteration under sub-lethal-dose aBL irradiation. At present, the latter possibility remains speculative and requires experimental evidence such as transcriptomics and RT-PCR in future studies. Collectively, the data indicate aBL-induced oxidative stress selectively alters membrane lipid composition, particularly targeting unsaturated phospholipids such as PE and PG. These changes may compromise membrane function, providing a plausible explanation for increased OM permeability and eventual bacterial inactivation.

**Table 2B:** 

Lipidion	Ratio of lipid intensity		
	0 j/cm^2^	15 j/cm^2^	30 j/cm^2^
CL(16:0-16:1-16:0-18:1)	1.000	1.241 ± 0.098	1.214 ± 0.042
PE(19:1-18:1)	1.000	1.559 ± 0.103	1.558 ± 0.047
PE(15:0-16:0)	1.000	2.024 ± 0.058	1.686 ± 0.098
PE(15:0-16:1)	1.000	1.127 ± 0.077	0.960 ± 0.078
PE(16:0-16:0)	1.000	1.012 ± 0.053	0.930 ± 0.149
PE(16:0-18:3)	1.000	1.032 ± 0.067	0.971 ± 0.062
PE(17:1-16:0)	1.000	1.019 ± 0.081	0.867 ± 0.046
PE(18:1-18:1)	1.000	1.751 ± 0.085	1.430 ± 0.037
PG(15:0-16:1)	1.000	1.182 ± 0.128	1.026 ± 0.129
PG(16:0-16:0)	1.000	1.339 ± 0.024	1.046 ± 0.042
PS(16:0-19:2)	1.000	1.820 ± 0.196	1.638 ± 0.044

#### 3.2.3 Changes of intracellular MDA, OM permeability and potassium leakage

**Figure 4:**
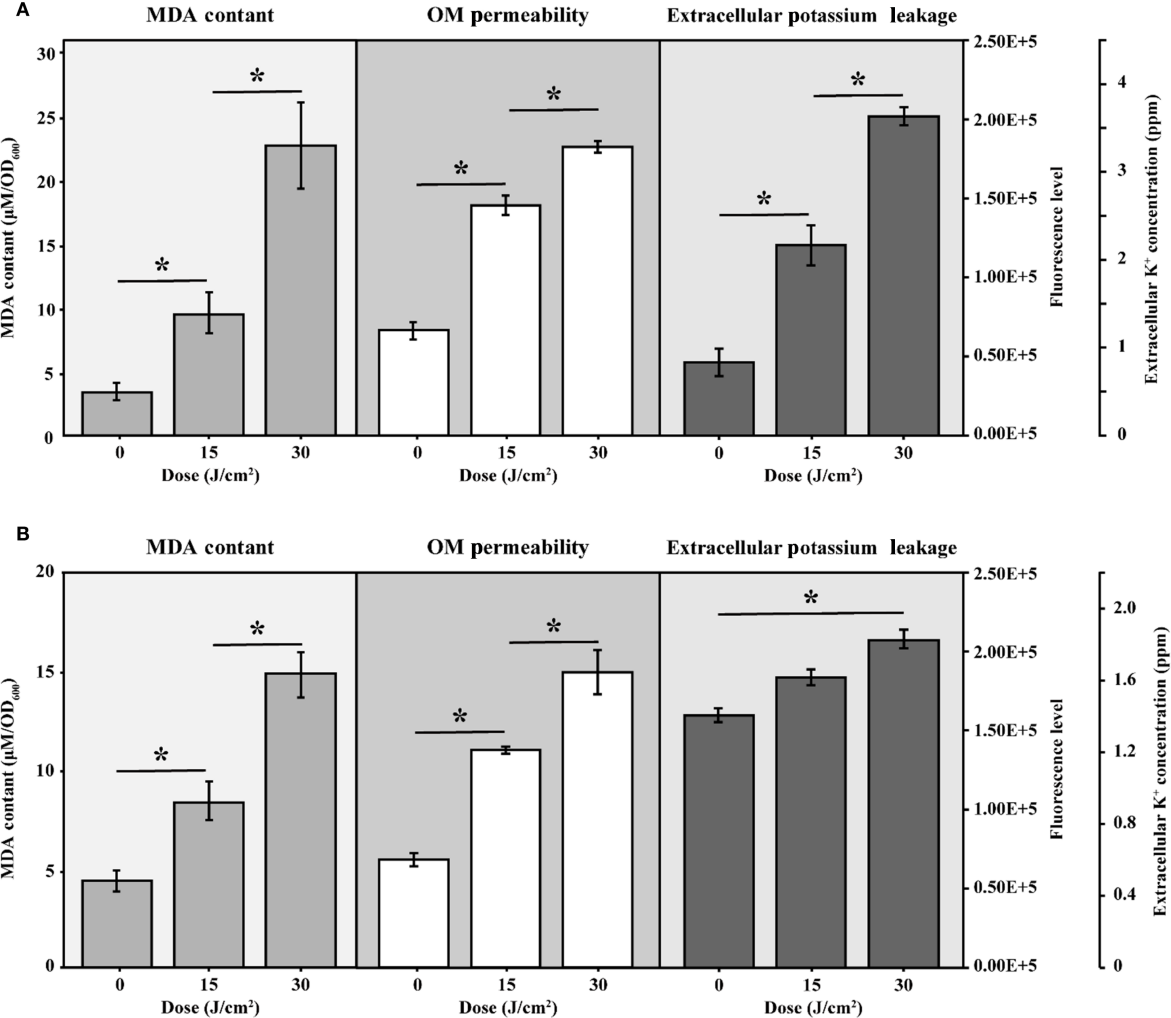
The intracellular MDA levels, OM permeability and extracellular potassium leakage of **(A)***E coli* W3110 and **(B)***S.* Typhimurium SL1344 during aBL irradiation. Intracellular MDA levels were measured as markers of lipid peroxidation, OM permeability was assessed using NPN uptake assays, and extracellular potassium leakage was measured by a flame photometer. Data represent the mean ± SD of three biological replicates, with significance set at *p* < 0.05.

MDA was widely recognized as a marker of oxidative stress. **Figure 4A** illustrated a progressive increase in MDA levels in cells subjected to aBL irradiation. With 15 J/cm² irradiation, the MDA content increased 2.85-fold, rising from 3.30 ± 0.56 µM/OD_600_ to 9.44 ± 1.66 µM/OD_600_ (*p* < 0.001). This increase was closely correlated with increase of ROS (**Supplementary Figure S4**), similar to our previous report (Wu et al., 2018). At aBL dose of 30 J/cm^2^, MDA level further increased to 22.64 ± 3.28 µM/OD_600_ (*p* < 0.001). OM permeability increased by approximately 65.20% and 130.40% at 15 and 30 J/cm², respectively, compared to the untreated control (**Figure 4A**). Besides, the extracellular potassium level also rose from 0.82 × 10^5^ ppm at 0 J/cm² to 1.53 × 10^5^ ppm at 15 J/cm², and further increased to 2.03 × 10^5^ ppm at 30 J/cm² (*p* < 0.05). The steady increase indicated substantial leakage of cytoplasmic potassium, supporting the reduction of OM compactness.

The progressive rise in MDA levels indicates enhanced lipid peroxidation within *E. coli*. These lipid disturbances most likely contribute to membrane damage and eventual bacterial inactivation as evidenced by the increased OM permeability, as determined by the NPN uptake assay, following aBL irradiation. The following section provides a detailed discussion of the alterations in unsaturated lipids.

#### 3.2.4 The impact of aBL on changes of unsaturated lipids

During aBL irradiation process, the unsaturated lipids exhibited significantly variability (**Table 1**). Lipids showing a stable increase mainly contained saturated fatty acid chains such as 16:0, 18:0, and 18:1, whereas those displaying a decrease were primarily composed of unsaturated chains such as 16:1, 17:1, and 18:1. Notably, unsaturated PE class became dominant. Among the various lipid classes, PE and PG exhibited the most significant changes under aBL irradiation. Among the 12 PE species that gradually declined, only PE(12:0-14:0) was saturated, while 4 species were mono-unsaturated, and 7 species were poly-unsaturated. Previous studies on PDT revealed that PE molecules with C16:1, C18:1, and C18:2 fatty acyl chains were particularly susceptible to ROS induced by photosensitizers (Alves et al., 2013b). In this study, PE, characterized by a high concentration of unsaturated double bonds, appeared more vulnerable to oxidative stress from aBL irradiation.

A similar result was also obtained for PG. All PG species showing decreasing trends were unsaturated, including PG(17:1-16:0), PG(16:0-18:1), and PG(19:1-16:0). The decline in unsaturated lipids, particularly PE and PG, was closely related to oxidative stress during aBL illumination, as demonstrated in various microbes in previous studies (Chu et al., 2019; Hu et al., 2021a; Wu et al., 2018).

**Figure 5:**
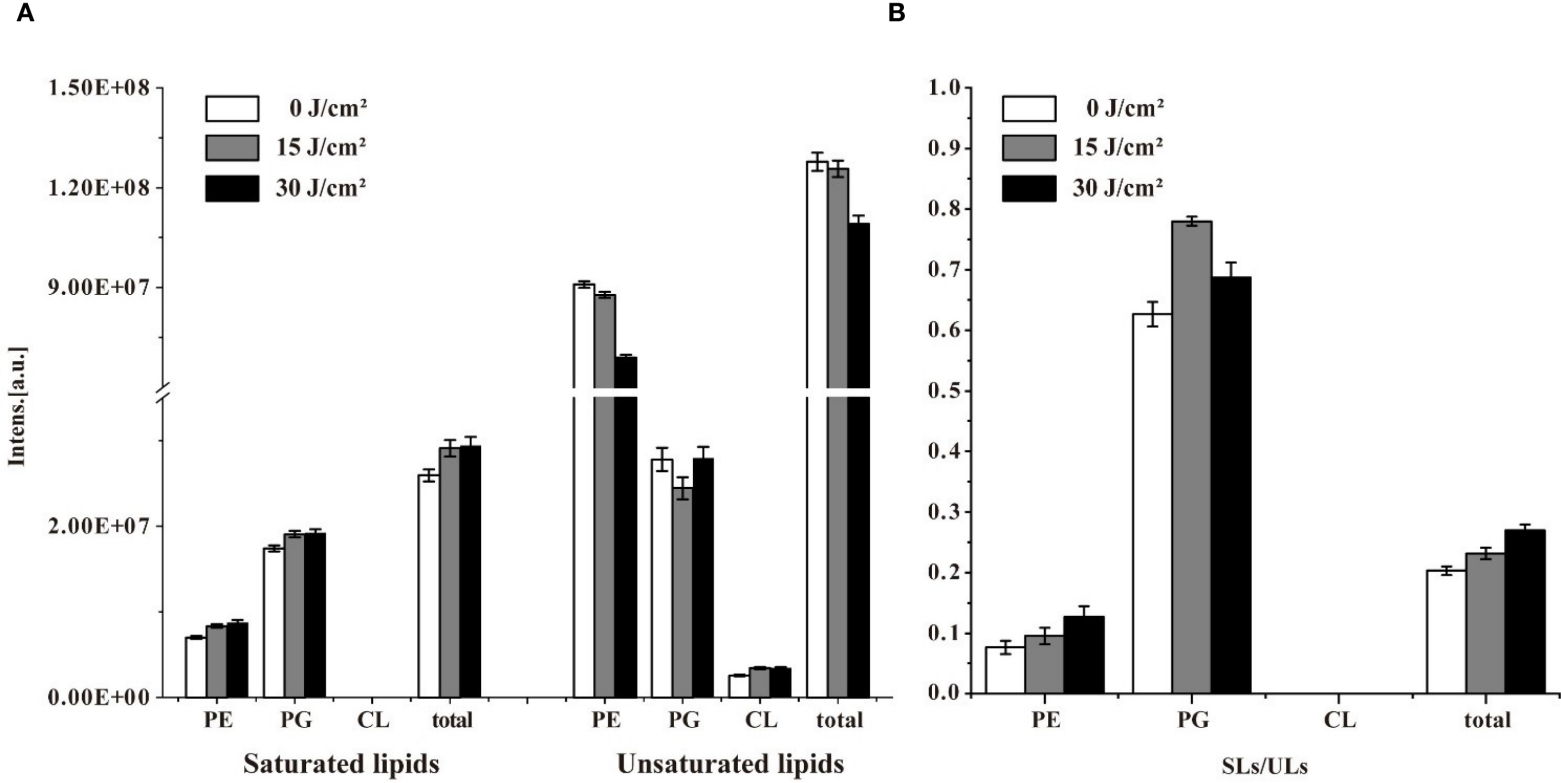
The intensity of saturated lipids (SLs) and unsaturated lipids (ULs), and their ratio (SLs/ULs) in in *E. coli* W3110 under different aBL doses. **(A)** Changes in the abundance of SLs and ULs were analyzed after exposure to 0, 15, and 30 J/cm^2^. **(B)** The SLs/ULs ratio reflected the degree of unsaturation in membrane lipids.

Changes in the abundance of unsaturated lipids were also detected. As shown in **Figure 5A**, the intensity of total saturated lipids increased after 15 J/cm^2^ and maintained stably when the irradiation was extended to 30 J/cm^2^. Notably, no saturated CL was detected during aBL treatment. Conversely, the intensity of unsaturated lipids decreased along with aBL irradiation. Unsaturated PE decreased by 12% and 24% at 15 and 30 J/cm^2^, respectively. However, unsaturated PG initially declined but later increased, and unsaturated CL showed an upward increase. CL was worth researching since it has been reported to induce apoptosis in prokaryotic cells (Mileykovskaya and Dowhan, 2009). It was postulated that CL play a critical role in aBL-induced bacterial death. Besides, the ratio of saturated to unsaturated lipids (SLs/ULs) was used to assess the degree of unsaturation in membrane lipids, with a higher SLs/ULs ratio indicating a reduced proportion of unsaturated membrane lipids. The shift from unsaturated to saturated lipid species, particularly in PE and PG, indicating fluctuations of membrane lipids. Lipids in biological membranes are thought to be functionally organized, attributed to their chemical diversity and the stoichiometry, i. e. their lipid composition (Melero and Jimenez-Rojo, 2023; Melero and Jimenez-Rojo, 2023). From a perspective of lipidomics, the study is the first to reveal changes in lipids profile and their intensity following aBL treatment, which critically contribute to cell membrane dysfunction, such as but not limited to the increased OM permeability.

As shown in **Figure 5B**. The SLs/ULs ratio for total lipids, particularly PE, progressively increased during aBL irradiation, consistent with the trends observed in **Figure 5A**. The observed increase in saturated lipids and concurrent decrease in unsaturated lipid species suggest a shift in membrane composition, since the membrane composition of cells belonging to a single species is not constant and varies with environmental conditions (Dowhan, 2013; Sohlenkamp and Geiger, 2016). Our study revealed that aBL irradiation significantly alters the composition and content of lipids in *E. coli*, particularly affecting unsaturated lipid species. Similar to that exposed to abiotic stress, the lipids modifications upon aBL illumination are not restricted to the fatty acid moieties but also occur at the headgroup level. The effects of aBL on *S*. Typhimurium lipids are discussed in the next section.

### 3.3 Influence of aBL on lipidomics of *S.* Typhimurium by LC-MS analysis

Similarly, in *S.* Typhimurium, 74 lipid species across 5 classes were identified using PLS-DA (**Supplementary Figure S1B**). PE and PG were the predominant lipids class, comprising 23 PE species and 12 PG species, all of which were identical to those found in *E. coli*, respectively. For other lipid classes such as LPE, PS and CL, there are 3, 6, 2 species similar to those in *E. coli*. These 46 lipids mentioned above maybe conserved in *E. coli* and *S.* Typhimurium.

Similar to its impact on *E. coli*, aBL irradiation induced notable changes in the lipidome of *S.* Typhimurium. All lipids were listed in **Supplementary Table S3**, and some lipids were demonstrated in **Supplementary Table S4**. Following exposure to 15 J/cm^2^ aBL treatment, changes were observed in the types and intensities of PE, PG, and CL (**Figure 3B**). Increasing the dose to 30 J/cm² further altered the lipid profile and content, as detailed below.

**Figure 6:**
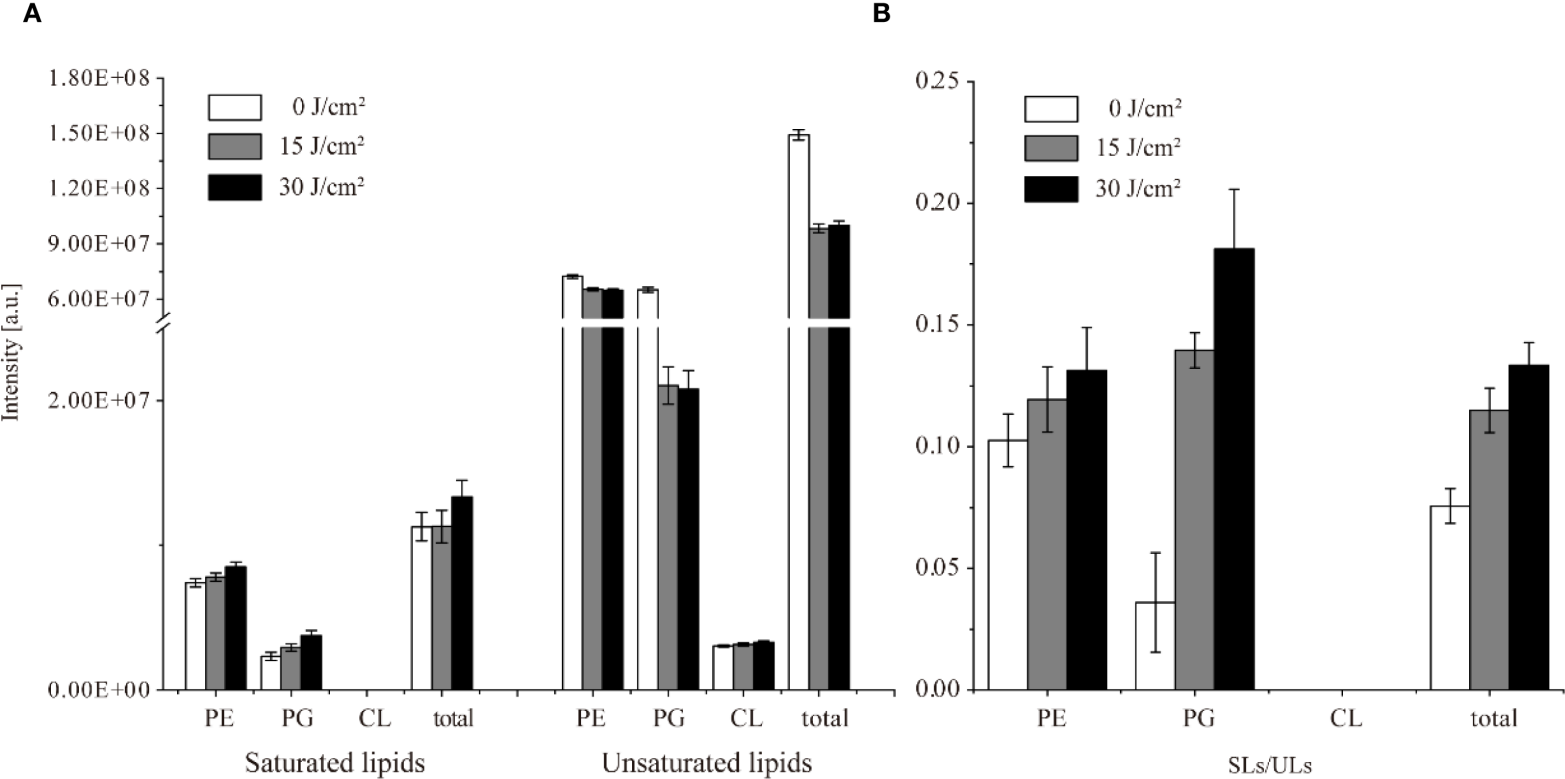
The intensity of saturated lipids (SLs) and unsaturated lipids (ULs), and their ratio (SLs/ULs) in *S.* Typhimurium SL1344 under different aBL doses. Changes in the abundance of SLs and ULs were analyzed after exposure to 0, 15, and 30 J/cm². The SLs/ULs ratio reflected the degree of unsaturation in membrane lipids.

Under aBL irradiation, *S.* Typhimurium exhibited more variable lipid changes compared to *E. coli.*, with 18 species showing erratic lipid changes (**Figure 6**; **Table 4**). Eleven PE species initially decreased at a 15 J/cm^2^ dose, and subsequently increased at 30 J/cm^2^. Comparatively, the changes in PE lipids showed a similar trend in both *E. coli* and *S.* Typhimurium (**Tables 2A, 2B**). However, in S. Typhimurium, a notable increase in saturated lipids and a decline in unsaturated lipids were observed, particularly within the PE class (**Figure 6**; **Table 5**).

**Table 3:** Two distinct patterns of specific lipid species in *S.* Typhimurium SL1344 upon aBL treatment: **(A)** initial decrease followed by increase, and **(B)** initial increase followed by decrease.

Lipidion	Ratio		
	0 j/cm^2^	15 j/cm^2^	30 j/cm^2^
CL (18:1-18:1-16:0-18:1)	1	0.903 ± 0.085	1.071 ± 0.077
PE (15:0-16:0)	1	0.994 ± 0.056	1.075 ± 0.077
PE (15:0-18:1)	1	0.833 ± 0.092	0.838 ± 0.068
PE (16:0-14:1)	1	0.952 ± 0.031	1.276 ± 0.079
PE (16:0-18:1)	1	0.841 ± 0.088	0.956 ± 0.155
PE (16:0-18:2)	1	0.858 ± 0.135	0.944 ± 0.065
PE (16:1-16:1)	1	0.953 ± 0.209	1.133 ± 0.158
PE (17:1-16:1)	1	0.919 ± 0.097	0.929 ± 0.079
PE (18:0-18:0)	1	0.882 ± 0.074	0.991 ± 0.068
PE (18:1-18:2)	1	0.888 ± 0.001	0.959 ± 0.060
PE (19:1-16:0)	1	0.841 ± 0.065	1.163 ± 0.132
PE (19:1-18:0)	1	0.830 ± 0.019	1.092 ± 0.090
PG (16:0-12:0)	1	0.890 ± 0.162	1.184 ± 0.067
PG (16:0-14:0)	1	0.737 ± 0.169	1.298 ± 0.091
PG (16:0-16:0)	1	0.831 ± 0.056	0.975 ± 0.008
PG (16:0-18:1)	1	0.898 ± 0.053	1.455 ± 0.038
PG (16:1-18:1)	1	0.936 ± 0.046	1.075 ± 0.051
PG (17:1-14:0)	1	0.905 ± 0.071	0.968 ± 0.084
PG (17:1-19:1)	1	0.923 ± 0.080	0.958 ± 0.030
PG (18:0-16:0)	1	0.804 ± 0.040	1.057 ± 0.033
PG (19:1-16:0)	1	0.531 ± 0.050	0.642 ± 0.049
PG (19:1-17:0)	1	0.801 ± 0.088	0.836 ± 0.124
PG (19:1-18:1)	1	0.480 ± 0.146	0.699 ± 0.087
PS (16:0-19:2)	1	0.831 ± 0.093	0.970 ± 0.146
PS (16:1-19:2)	1	0.864 ± 0.124	0.894 ± 0.100

LipidIon	Ratio		
	0 J/cm^2^	15 J/cm^2^	30 J/cm^2^
CL (17:1-18:1-16:0-16:0)	1	1.285 ± 0.098	1.117 ± 0.039
LPE (17:1)	1	1.241 ± 0.136	1.159 ± 0.076
PE (16:0-16:0)	1	1.020 ± 0.046	0.781 ± 0.061
PE (16:1-12:0)	1	1.089 ± 0.086	1.038 ± 0.155
PE (17:1-12:0)	1	1.021 ± 0.057	0.879 ± 0.077
PE (17:1-17:1)	1	1.095 ± 0.085	0.982 ± 0.086
PE (17:1-18:1)	1	1.394 ± 0.090	0.961 ± 0.076
PE (18:1-18:1)	1	1.155 ± 0.066	0.866 ± 0.099
PE (19:1-17:0)	1	1.304 ± 0.065	1.153 ± 0.045
PE (20:0-18:1)	1	1.085 ± 0.052	1.079 ± 0.077
PG (16:1-14:0)	1	1.056 ± 0.088	1.040 ± 0.094
PG (17:1-15:0)	1	1.208 ± 0.046	1.109 ± 0.004
PG (17:1-16:1)	1	1.053 ± 0.043	1.030 ± 0.029
PG (20:1-18:1)	1	1.045 ± 0.007	0.888 ± 0.111
PS (17:1-19:2)	1	1.099 ± 0.098	0.977 ± 0.027
PS (17:1-21:3)	1	1.243 ± 0.065	0.731 ± 0.071
PS (18:1-21:3)	1	1.202 ± 0.091	1.193 ± 0.087
PS (19:2-14:0)	1	1.252 ± 0.074	1.132 ± 0.051

**Table 5:** The continuous changes in specific lipid species of *S.* Typhimurium SL1344 during aBL treatment.

Dynamic trends of lipid species under different conditions	Lipid species	Adduct	Formula	M/z
Lipid species showing continuous increase	CL (16:0-16:1-16:0-18:1)	[M − 2H]^2-^	C_75_ H_140_ O_17_ P_2_	687.478839
	CL (18:1-16:0-16:0-18:1)	[M − 2H]^2-^	C_77_ H_144_ O_17_ P_2_	701.494489
	CL (18:1-16:1-16:0-18:1)	[M − 2H]^2-^	C_77_ H_142_ O_17_ P_2_	700.486664
	CL (19:1-18:1-16:0-16:0)	[M − 2H]^2-^	C_78_ H_146_ O_17_ P_2_	708.502314
	PE (16:0-12:0)	[M − H]^-^	C_33_ H_65_ O_8_ N_1_ P_1_	634.445331
	PE (16:0-14:0)	[M − H]^-^	C_35_ H_69_ O_8_ N_1_ P_1_	660.460981
	PE (15:0-16:1)	[M − H]^-^	C_36_ H_69_ O_8_ N_1_ P_1_	674.476631
	PE (16:0-17:0)	[M − H]^-^	C_38_ H_75_ O_8_ N_1_ P_1_	704.523581
	PE (18:0-16:0)	[M − H]^-^	C_39_ H_77_ O_8_ N_1_ P_1_	718.539231
	PE (16:0-20:1)	[M − H]^-^	C_41_ H_79_ O_8_ N_1_ P_1_	744.555605
	PE (18:0-18:1)	[M − H]^-^	C_41_ H_79_ O_8_ N_1_ P_1_	744.555653
	PE (19:1-19:1)	[M − H]^-^	C_43_ H_81_ O_8_ N_1_ P_1_	770.570531
	PG (16:1-16:1)	[M − H]^-^	C_38_ H_70_ O_10_ N_0_ P_1_	717.471212
	PG (16:0-16:1)	[M − H]^-^	C_38_ H_72_ O_10_ N_0_ P_1_	719.487146
	PG (17:1-18:1)	[M − H]^-^	C_41_ H_76_ O_10_ N_0_ P_1_	759.518162
	PS (16:0p-17:1)	[M − H]^-^	C_39_ H_73_ O_9_ N_1_ P_1_	730.502846
Lipid species showing continuous decrease	PE (12:0-14:0)	[M − H]^-^	C_31_ H_61_ O_8_ N_1_ P_1_	606.4140305
	PE (15:0-14:0)	[M − H]^-^	C_34_ H_67_ O_8_ N_1_ P_1_	648.4609805
	PE (18:1-12:0)	[M − H]^-^	C_35_ H_67_ O_8_ N_1_ P_1_	660.4609805
	PE (18:1-14:0)	[M − H]^-^	C_37_ H_71_ O_8_ N_1_ P_1_	688.4922805
	PE (17:1-17:1)	[M − H]^-^	C_39_ H_73_ O_8_ N_1_ P_1_	714.5079305
	PE (17:1-19:1)	[M − H]^-^	C_41_ H_77_ O_8_ N_1_ P_1_	742.5392305
	PE (19:1-18:1)	[M − H]^-^	C_42_ H_79_ O_8_ N_1_ P_1_	756.5548805
	PE (16:0-18:3)	[M − H]^-^	C_39_ H_71_ O_8_ N_1_ P_1_	712.4922805
	PG (16:0-18:2)	[M − H]^-^	C_40_ H_74_ O_10_ N_0_ P_1_	745.5025115
	PG (18:1-18:1)	[M − H]^-^	C_42_ H_78_ O_10_ N_0_ P_1_	773.5338115
	PG (19:1-18:0)	[M − H]^-^	C_43_ H_82_ O_10_ N_0_ P_1_	789.5651115
	PG (19:1-19:1)	[M − H]^-^	C_44_ H_82_ O_10_ N_0_ P_1_	801.5651115
	PS (17:1-20:3)	[M − H]^-^	C_43_ H_75_ O_10_ N_1_ P_1_	796.5134105

**Figure 7:**
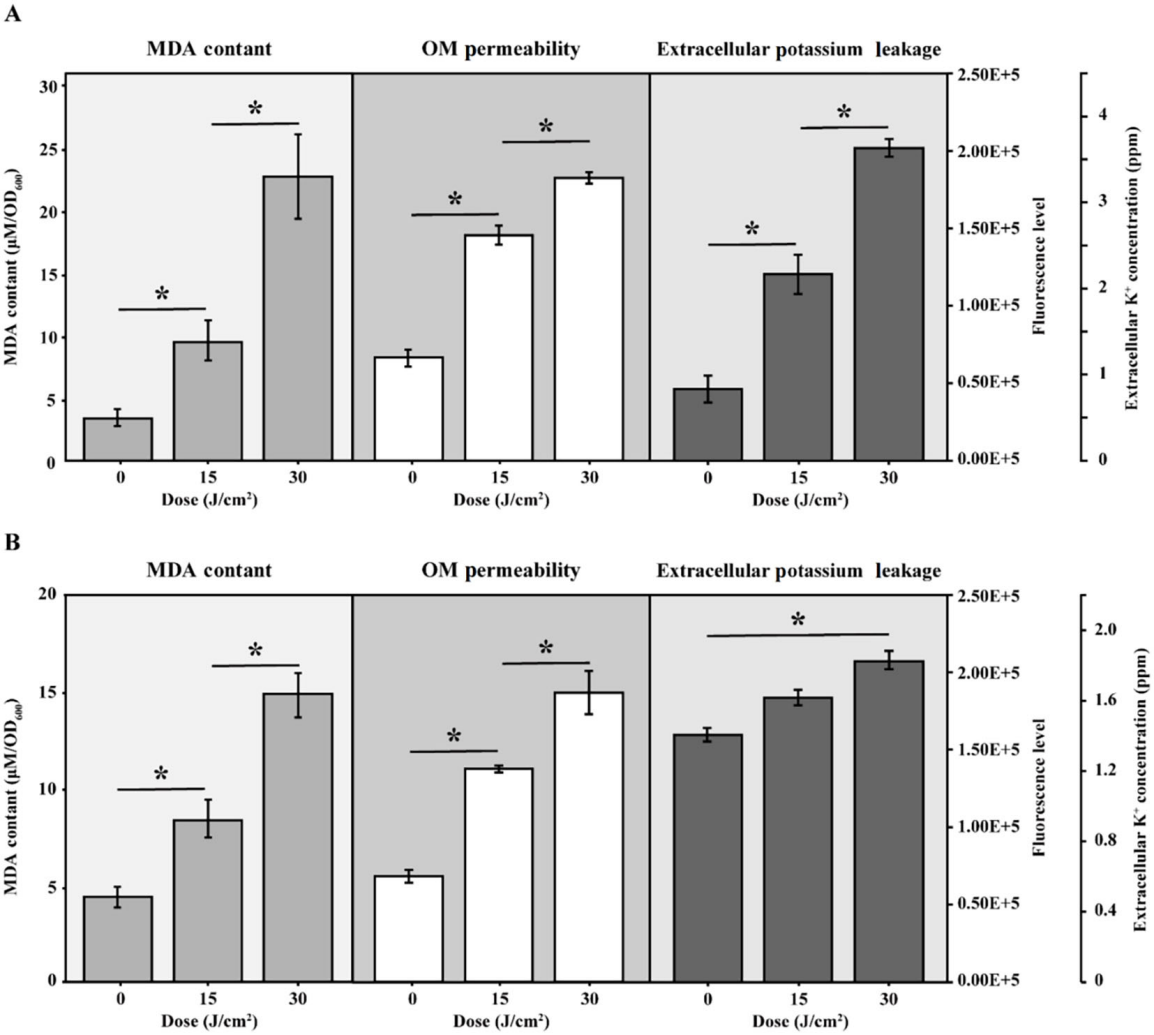
Intensity of lipids under different aBL irradiation doses in *S.* Typhimurium SL1344 **(A)** Total lipid intensity changes under 0, 15, and 30 J/cm² aBL. There existed significant differences between different samples (*p* < 0.05). “a” meant significant differences between samples under 0 and 15 J/cm², “b” meant that under 0 and 30 J/cm², and “c” meant that under 15 and 30 J/cm²; **(B, C)** Representative lipids exhibiting discontinuous changes in the intensity under different aBL doses.

More PG species were detected in *S.* Typhimurium than in *E. coli*, although fewer of them exhibited changes during aBL irradiation (**Figure 7A**). Seven PG species exhibited continuous increases or decreases, whereas 16 PG species displayed erratic patterns. Additionally, CL species in *S.* Typhimurium differed from those found in *E. coli.* Notably, CL(18:1-16:1-16:0-18:1) increased in *S.* Typhimurium but decreased in *E. coli*.

However, the overall extent of lipid changes, particularly in PE and PG species, was less pronounced and less consistent in *S.* Typhimurium compared to *E. coli*, as fewer lipid species exhibited statistically significant or directional changes across irradiation doses. This raises the question of whether such differences in lipid fluctuation correlate with *S.* Typhimurium’s lower sensitivity to aBL irradiation.

Next, MDA level, OM permeability and potassium leakage in *S.* Typhimurium exposed to aBL were determined. As illustrated in **Figure 4B**, the MDA levels increased from 4.37 ± 0.71 µM/OD_600_ before aBL irradiation to 8.37 ± 0.97 µM/OD_600_ after 15 J/cm^2^, then declined to 4.77 ± 1.69 µM/OD_600_ at after 30 J/cm^2^ dose. Compared to *E. coli*, where MDA levels increased to 22.64 ± 3.28 µM/OD_600_ at 30 J/cm^2^, *S.* Typhimurium exhibited lower MDA levels (*p* < 0.001). Besides, OM permeability in *S.* Typhimurium increased markedly, rising by 101.65% at 15 J/cm^2^ and reaching 174.38% at 30 J/cm^2^ (**Figure 6B**), likely due to the oxidative stress-induced alterations in membrane composition (*p* < 0.001). Additionally, although *S.* Typhimurium exhibited high background leakage, it still showed a significant increase along with aBL irradiation.

This study demonstrates the profound impact of aBL irradiation on the lipidomic profiles of *S.* Typhimurium and *E. coli*. Our findings reveal substantial diversity and variability in lipid composition across sublethal aBL irradiations. Notably, we observed significant alteration membrane lipids—particularly unsaturated species—likely driven by aBL-induced oxidative stress. These insights are critical for deciphering the bactericidal mechanisms of aBL and uncovering the molecular basis of strain-specific difference in aBL sensitivity.

## Data Availability

The raw data supporting the conclusions of this article will be made available by the authors, without undue reservation.
