# Supplementary material for: The impact of sublethal antimicrobial blue light on lipidomic changes of Escherichia coli and Salmonella Typhimurium
Source: Front Cell Infect Microbiol. 2025 Nov 26;15:1612638. doi: 10.3389/fcimb.2025.1612638 (PMC12689863; doi:10.3389/fcimb.2025.1612638)
Supplement: Supplementary file 1 [file DataSheet1.pdf]

# *Supplementary Material*

## 1 Supplementary Figures and Tables

### 1.1 Supplementary Figures

**Fig. S1.** PLS-DA score plot derived from the UHPLC-ESI-MS data of (A) *E. coli* and (B) *S.* typhimurium SL1344. Abbreviations: PC1, principal component 1; PC2, principal component 2; PC3, principal component 3

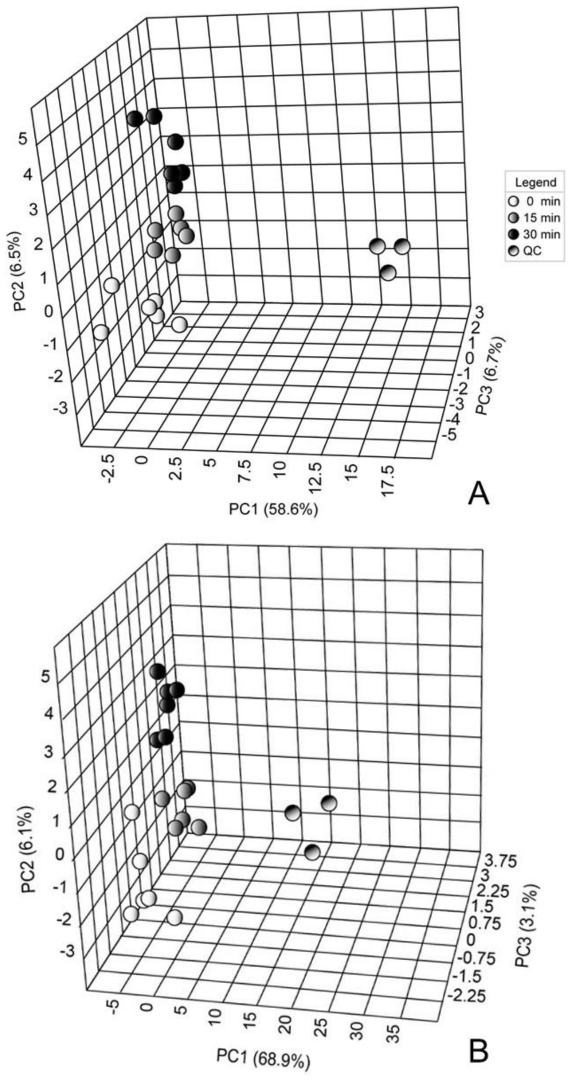

**Fig. S2.** Mass spectra of the lipid extracts from *E. coli* W3110

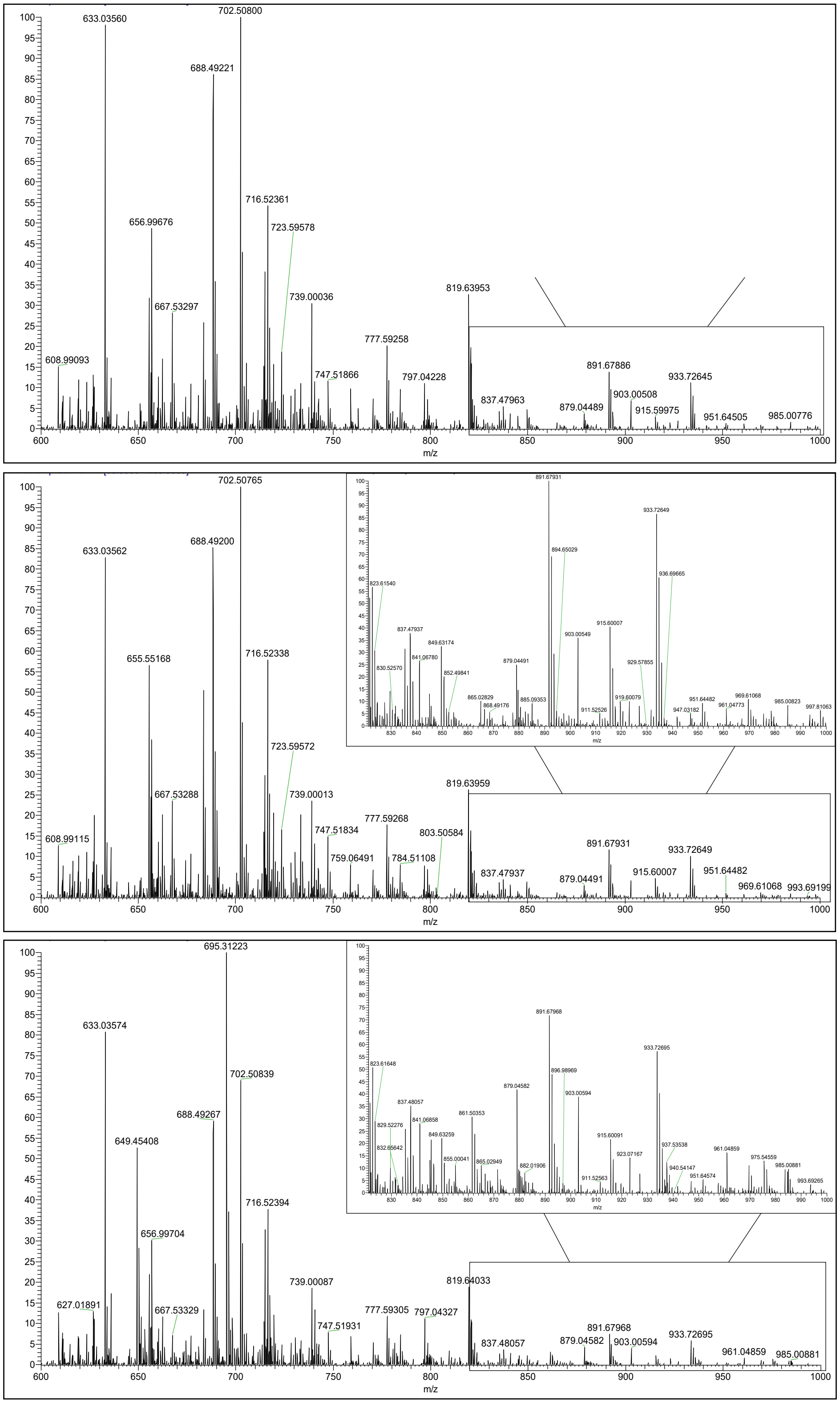

**Fig. S3.** Elucidation of fragmentation patterns of glycerophospholipids with representative PE(17:0-16:0) and PG(16:0-18:1). (A) MS/MS spectra of PE (17:0-16:0) under negative modes; (B) MS/MS spectra of PG(16:0-18:1) under negative modes

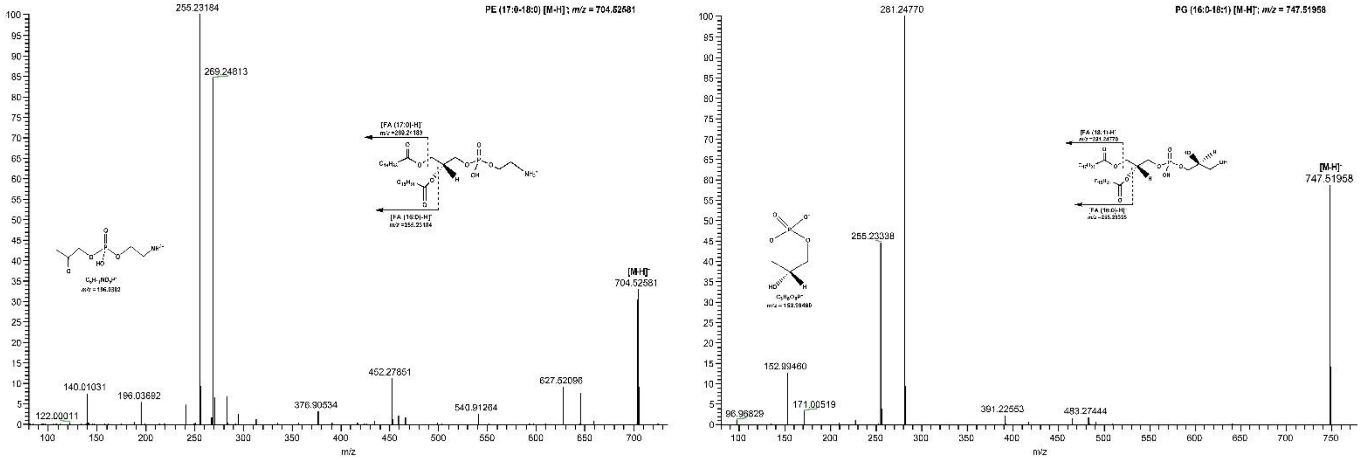


A B

**Fig. S4.** Blue light irradiation induced ROS accumulation in *E. coli* W3110 and *S.* Typhimurium SL1344. Bacteria were exposed to indicated doses (0, 15, and 30 J/cm²), and the intracellular ROS levels were quantified using a fluorescence-based DCFH-DA assay. Fluorescence intensity represented the relative ROS levels in each sample. Data are presented as mean ± SD from three independent experiments. * indicated statistically significant difference compared to the corresponding 0 J/cm² group of each strain ($p < 0.05$, Student's t-test)

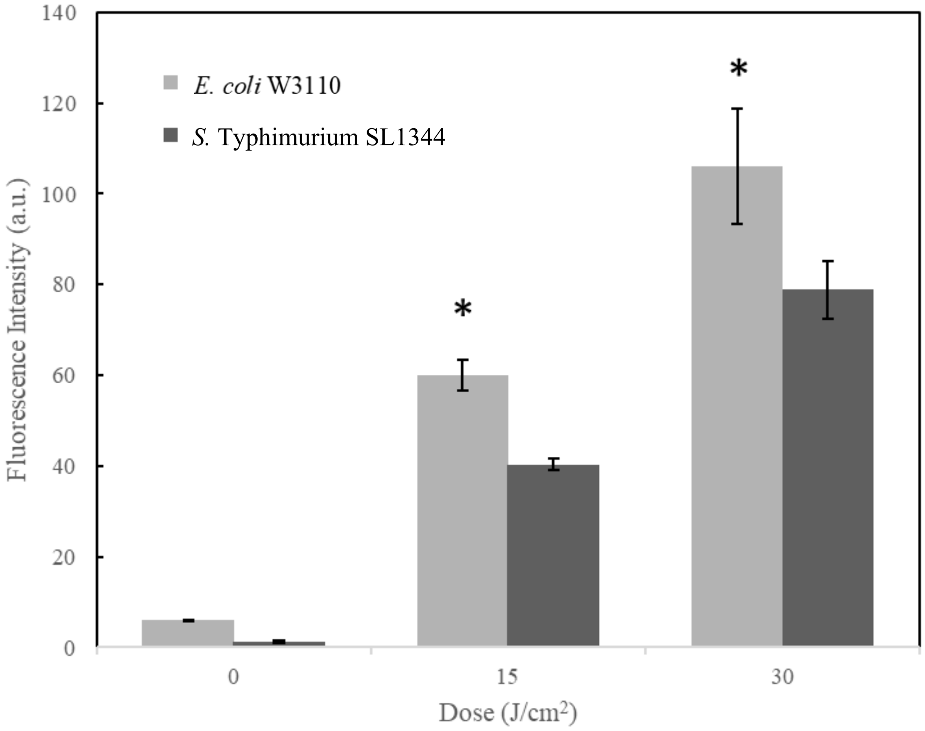

## 1.2 Supplementary Tables

**Table S1** List of all lipids identified in *E. coli* W3110 during aBL illumination

(Please refer to Excel file 1 for the raw data)

**Table S2** List of main lipids identified in *E. coli* W3110 during aBL illumination

| 0 J/cm$^2$ | | 15 J/cm$^2$ | | 30 J/cm$^2$ | |
|---|---|---|---|---|---|
| Class | LipidIon | Class | LipidIon | Class | LipidIon |
| CL | CL(14:0-16:1-16:0-16:0) | CL | CL(14:0-16:1-16:0-16:0) | CL | CL(16:0-16:1-16:0-16:0) |
| CL | CL(16:0-16:0-16:0-16:1) | CL | CL(16:0-16:1-16:0-16:0) | CL | CL(17:1-16:1-16:0-16:0) |
| CL | CL(16:0-16:1-16:0-16:1) | CL | CL(16:0-16:1-16:0-16:1) | CL | CL(16:0-16:1-16:0-18:1) |
| CL | CL(16:0-16:1-16:0-18:1) | CL | CL(17:1-16:1-16:0-16:0) | CL | CL(18:1-16:1-16:0-16:1) |
| CL | CL(17:1-16:1-16:0-16:0) | CL | CL(16:0-16:1-16:0-18:1) | CL | CL(17:1-18:1-16:0-16:0) |
| CL | CL(17:1-18:1-16:0-16:0) | CL | CL(18:1-16:1-16:0-16:1) | CL | CL(18:1-18:1-16:0-16:0) |
| CL | CL(18:1-16:1-16:0-18:1) | CL | CL(17:1-18:1-16:0-16:0) | CL | CL(18:1-16:1-16:0-18:1) |
| CL | CL(18:1-18:1-16:0-16:0) | CL | CL(18:1-18:1-16:0-16:0) | CL | CL(16:0-16:1-16:0-16:0) |
| CL | CL(18:1-16:1-16:0-16:1) | CL | CL(18:1-16:1-16:0-18:1) | LPE | LPE(16:0) |
| CL | CL(24:7-16:0-16:0-20:1) | LPE | LPE(16:0) | LPE | LPE(16:1) |
| LPE | LPE(16:0) | LPE | LPE(16:1) | LPE | LPE(17:1) |
| LPE | LPE(16:1) | LPE | LPE(17:1) | LPE | LPE(18:1) |
| LPE | LPE(17:1) | LPE | LPE(18:1) | PE | PE(12:0-14:0) |
| LPE | LPE(18:1) | PE | PE(12:0-14:0) | PE | PE(15:0-14:0) |
| PE | PE(12:0-14:0) | PE | PE(15:0-14:0) | PE | PE(15:0-16:0) |
| PE | PE(15:0-14:0) | PE | PE(15:0-16:0) | PE | PE(15:0-16:1) |
| PE | PE(15:0-16:0) | PE | PE(15:0-16:1) | PE | PE(16:0-12:0) |
| PE | PE(15:0-16:1) | PE | PE(16:0-12:0) | PE | PE(16:0-14:0) |
| PE | PE(16:0-12:0) | PE | PE(16:0-14:0) | PE | PE(16:0-16:0) |
| PE | PE(16:0-14:0) | PE | PE(16:0-16:0) | PE | PE(16:0-17:0) |
| PE | PE(16:0-16:0) | PE | PE(16:0-17:0) | PE | PE(16:0-18:1) |
| PE | PE(16:0-17:0) | PE | PE(16:0-18:1) | PE | PE(16:0-18:3) |
| PE | PE(16:0-18:1) | PE | PE(16:0-18:3) | PE | PE(16:1-12:0) |
| PE | PE(16:0-18:3) | PE | PE(16:1-12:0) | PE | PE(16:1-16:1) |
| PE | PE(16:1-12:0) | PE | PE(16:1-16:1) | PE | PE(16:1-18:1) |
| PE | PE(16:1-14:1) | PE | PE(16:1-18:1) | PE | PE(17:1-16:0) |
| PE | PE(16:1-16:1) | PE | PE(17:1-16:0) | PE | PE(17:1-16:1) |
| PE | PE(16:1-18:1) | PE | PE(17:1-16:1) | PE | PE(17:1-17:1) |
| PE | PE(17:1-12:0) | PE | PE(17:1-17:1) | PE | PE(17:1-18:1) |
| PE | PE(17:1-16:0) | PE | PE(17:1-18:1) | PE | PE(17:1-19:1) |
| PE | PE(17:1-16:1) | PE | PE(17:1-19:1) | PE | PE(18:0-16:0) |
| PE | PE(17:1-17:1) | PE | PE(18:0-16:0) | PE | PE(18:0-18:1) |
| PE | PE(17:1-18:1) | PE | PE(18:0-18:1) | PE | PE(18:1-12:0) |
| PE | PE(17:1-19:1) | PE | PE(18:1-12:0) | PE | PE(18:1-14:0) |
| PE | PE(18:0-16:0) | PE | PE(18:1-14:0) | PE | PE(18:1-18:1) |
| PE | PE(18:0-18:1) | PE | PE(18:1-18:1) | PE | PE(19:1-16:0) |
| PE | PE(18:1-12:0) | PE | PE(19:1-16:0) | PE | PE(19:1-18:1) |
| PE | PE(18:1-14:0) | PE | PE(19:1-18:1) | dMePE | dMePE(12:0-14:0) |
| PE | PE(18:1-18:1) | PG | PG(15:0-16:0) | PG | PG(15:0-16:0) |
| PE | PE(19:1-16:0) | PG | PG(15:0-16:1) | PG | PG(15:0-16:1) |
| PE | PE(19:1-18:1) | PG | PG(16:0-12:0) | PG | PG(16:0-12:0) |
| PG | PG(15:0-16:0) | PG | PG(16:0-14:0) | PG | PG(16:0-14:0) |
| PG | PG(15:0-16:1) | PG | PG(16:0-14:1) | PG | PG(16:0-14:1) |
| PG | PG(16:0-12:0) | PG | PG(16:0-16:0) | PG | PG(16:0-16:0) |
| PG | PG(16:0-14:0) | PG | PG(16:0-16:1) | PG | PG(16:0-16:1) |
| PG | PG(16:0-14:1) | PG | PG(16:0-18:1) | PG | PG(16:0-18:1) |
| PG | PG(16:0-16:0) | PG | PG(16:0-18:2) | PG | PG(16:0-18:2) |

| | | | | | |
|---|---|---|---|---|---|
| PG | PG(16:0-16:1) | PG | PG(16:1-14:0) | PG | PG(16:1-14:0) |
| PG | PG(16:0-18:1) | PG | PG(16:1-16:1) | PG | PG(16:1-16:1) |
| PG | PG(16:0-18:2) | PG | PG(16:1-18:1) | PG | PG(16:1-18:1) |
| PG | PG(16:1-14:0) | PG | PG(17:1-14:0) | PG | PG(17:1-14:0) |
| PG | PG(16:1-16:1) | PG | PG(17:1-16:0) | PG | PG(17:1-16:0) |
| PG | PG(16:1-18:1) | PG | PG(17:1-16:1) | PG | PG(17:1-16:1) |
| PG | PG(17:1-14:0) | PG | PG(17:1-18:1) | PG | PG(17:1-18:1) |
| PG | PG(17:1-16:0) | PG | PG(18:0-16:0) | PG | PG(18:0-16:0) |
| PG | PG(17:1-16:1) | PG | PG(18:0-18:1) | PG | PG(18:0-18:1) |
| PG | PG(17:1-18:1) | PG | PG(18:1-18:1) | PG | PG(18:1-18:1) |
| PG | PG(18:0-16:0) | PG | PG(19:1-16:0) | PG | PG(19:1-16:0) |
| PG | PG(18:0-18:1) | PS | PS(16:0-8:0) | PS | PS(16:0-8:0) |
| PG | PG(18:1-18:1) | PS | PS(16:0-19:2) | PS | PS(16:0p-17:1) |
| PG | PG(19:1-16:0) | PS | PS(16:0p-17:1) | PS | PS(16:0-19:2) |
| PS | PS(16:0-8:0) | PS | PS(16:1-17:2) | PS | PS(16:1-19:2) |
| PS | PS(16:0-19:2) | PS | PS(16:1-19:2) | PS | PS(16:1-19:3) |
| PS | PS(16:1-19:2) | PS | PS(16:1-19:3) | PS | PS(17:1-19:2) |
| PS | PS(16:1-19:3) | PS | PS(17:1-17:2) | PS | PS(17:1-20:3) |
| PS | PS(17:1-19:2) | PS | PS(17:1-19:2) | PS | PS(18:1-19:2) |
| PS | PS(17:1-20:3) | PS | PS(17:1-20:3) | PS | PS(18:1-21:3) |
| PS | PS(18:1-21:3) | PS | PS(18:1-21:3) | PS | PS(19:2-14:0) |
| PS | PS(19:2-14:0) | PS | PS(19:2-14:0) | PS | PS(19:2-14:1) |
| PS | PS(19:2-14:1) | | | | |

**Table S3** List of all lipids identified in *S.* typhimurium SL1344 during aBL illumination

(Please refer to Excel file 2 for the raw data)

**Table S4** List of main lipids identified in *S.* typhimurium SL1344 during aBL illumination

| 0 J/cm$^2$ | | 15 J/cm$^2$ | | 30 J/cm$^2$ | |
|---|---|---|---|---|---|
| Class | LipidIon | Class | LipidIon | Class | LipidIon |
| CL | CL(16:0/16:1/16:0/18:1) | CL | CL(17:1-18:1-16:0-16:0) | CL | CL(16:0-16:1-16:0-18:1) |
| CL | CL(17:1/18:1/16:0/16:0) | CL | CL(17:1-18:1-16:0-18:1) | CL | CL(17:1-18:1-16:0-16:0) |
| CL | CL(17:1/18:1/16:0/18:1) | CL | CL(18:1-16:0-16:0-18:1) | CL | CL(17:1-18:1-16:0-18:1) |
| CL | CL(18:1/16:0/16:0/18:1) | CL | CL(18:1-16:1-16:0-16:0) | CL | CL(18:1-16:0-16:0-18:1) |
| CL | CL(18:1/18:1/16:0/18:1) | CL | CL(18:1-18:1-16:0-16:1) | CL | CL(18:1-18:1-16:0-16:1) |
| LPE | LPE(16:0) | CL | CL(18:1-18:1-16:0-18:1) | CL | CL(18:1-18:1-16:0-18:1) |
| LPE | LPE(17:1) | LPE | LPE(16:0) | LPE | LPE(16:0) |
| LPE | LPE(18:1) | LPE | LPE(17:1) | LPE | LPE(17:1) |
| PE | PE(12:0-14:0) | LPE | LPE(18:1) | LPE | LPE(18:1) |
| PE | PE(14:0-13:0) | PE | PE(14:0-13:0) | PE | PE(12:0-14:0) |
| PE | PE(15:0-16:1) | PE | PE(15:0-14:0) | PE | PE(14:0-13:0) |
| PE | PE(15:0-18:1) | PE | PE(15:0-16:0) | PE | PE(15:0-16:0) |
| PE | PE(16:0-9:0) | PE | PE(15:0-16:1) | PE | PE(15:0-16:1) |
| PE | PE(16:0-12:0) | PE | PE(15:0-18:1) | PE | PE(15:0-18:1) |
| PE | PE(16:0-14:0) | PE | PE(16:0-12:0) | PE | PE(16:0-12:0) |
| PE | PE(16:0-16:0) | PE | PE(16:0-14:0) | PE | PE(16:0-14:0) |
| PE | PE(16:0-17:0) | PE | PE(16:0-16:0) | PE | PE(16:0-16:0) |
| PE | PE(16:0-18:1) | PE | PE(16:0-17:0) | PE | PE(16:0-17:0) |
| PE | PE(16:0-18:3) | PE | PE(16:0-18:1) | PE | PE(16:0-18:1) |
| PE | PE(16:1-12:0) | PE | PE(16:0-18:3) | PE | PE(16:0-18:3) |
| PE | PE(16:1-14:0) | PE | PE(16:0-9:0) | PE | PE(16:1-12:0) |
| PE | PE(16:1-16:1) | PE | PE(16:1-12:0) | PE | PE(16:1-14:0) |
| PE | PE(16:1-18:1) | PE | PE(16:1-14:0) | PE | PE(16:1-16:1) |
| PE | PE(17:1-12:0) | PE | PE(16:1-16:1) | PE | PE(16:1-18:1) |
| PE | PE(17:1-16:1) | PE | PE(16:1-18:1) | PE | PE(17:1-12:0) |
| PE | PE(17:1-17:1) | PE | PE(17:0-18:1) | PE | PE(17:1-16:1) |
| PE | PE(17:1-18:1) | PE | PE(17:1-12:0) | PE | PE(17:1-17:1) |
| PE | PE(17:1-19:1) | PE | PE(17:1-16:1) | PE | PE(17:1-18:1) |
| PE | PE(18:0-16:0) | PE | PE(17:1-17:1) | PE | PE(17:1-19:1) |
| PE | PE(18:0-18:0) | PE | PE(17:1-18:1) | PE | PE(18:0-16:0) |
| PE | PE(18:0-18:1) | PE | PE(17:1-19:1) | PE | PE(18:0-18:0) |
| PE | PE(18:0-20:1) | PE | PE(18:0-16:0) | PE | PE(18:0-18:1) |
| PE | PE(18:1-12:0) | PE | PE(18:0-18:0) | PE | PE(18:0-20:1) |
| PE | PE(18:1-14:0) | PE | PE(18:1-12:0) | PE | PE(18:1-12:0) |
| PE | PE(18:1-18:1) | PE | PE(18:1-14:0) | PE | PE(18:1-14:0) |
| PE | PE(18:1-18:2) | PE | PE(18:1-18:1) | PE | PE(18:1-18:1) |
| PE | PE(19:1-16:0) | PE | PE(18:1-18:2) | PE | PE(18:1-18:2) |
| PE | PE(19:1-18:0) | PE | PE(19:1-17:0) | PE | PE(19:1-16:0) |
| PE | PE(19:1-18:1) | PE | PE(19:1-18:0) | PE | PE(19:1-18:0) |
| PE | PE(19:1-19:1) | PE | PE(19:1-18:1) | PE | PE(19:1-18:1) |
| PE | PE(20:1-18:0) | PE | PE(19:1-19:1) | PE | PE(19:1-19:1) |
| PE | PE(20:1-18:1) | PE | PE(20:0-18:1) | PE | PE(20:1-18:1) |

| | | | | | |
|---|---|---|---|---|---|
| PG | PG(16:0-12:0) | PG | PG(16:0-12:0) | PG | PG(16:0-12:0) |
| PG | PG(16:0-14:0) | PG | PG(16:0-14:0) | PG | PG(16:0-14:0) |
| PG | PG(16:0-16:0) | PG | PG(16:0-16:0) | PG | PG(16:0-16:0) |
| PG | PG(16:0-16:1) | PG | PG(16:0-16:1) | PG | PG(16:0-16:1) |
| PG | PG(16:0-18:1) | PG | PG(16:0-18:1) | PG | PG(16:0-18:1) |
| PG | PG(16:0-18:2) | PG | PG(16:0-18:2) | PG | PG(16:0-18:2) |
| PG | PG(16:1-14:0) | PG | PG(16:1-14:0) | PG | PG(16:1-14:0) |
| PG | PG(16:1-16:1) | PG | PG(16:1-16:1) | PG | PG(16:1-18:1) |
| PG | PG(16:1-18:1) | PG | PG(16:1-18:1) | PG | PG(17:1-14:0) |
| PG | PG(17:1-14:0) | PG | PG(17:1-14:0) | PG | PG(17:1-16:0) |
| PG | PG(17:1-16:0) | PG | PG(17:1-16:0) | PG | PG(17:1-16:1) |
| PG | PG(17:1-16:1) | PG | PG(17:1-16:1) | PG | PG(17:1-17:0) |
| PG | PG(17:1-17:0) | PG | PG(17:1-17:1) | PG | PG(17:1-17:1) |
| PG | PG(17:1-17:1) | PG | PG(17:1-18:1) | PG | PG(17:1-18:1) |
| PG | PG(17:1-18:1) | PG | PG(17:1-19:1) | PG | PG(17:1-19:1) |
| PG | PG(17:1-19:1) | PG | PG(18:0-16:0) | PG | PG(18:0-14:0) |
| PG | PG(18:0-16:0) | PG | PG(18:1-14:0) | PG | PG(18:0-16:0) |
| PG | PG(18:1-14:1) | PG | PG(18:1-18:1) | PG | PG(18:1-18:1) |
| PG | PG(18:1-18:1) | PG | PG(19:1-16:0) | PG | PG(19:1-16:0) |
| PG | PG(19:1-16:0) | PG | PG(19:1-17:0) | PG | PG(19:1-17:0) |
| PG | PG(19:1-17:0) | PG | PG(19:1-18:0) | PG | PG(19:1-18:0) |
| PG | PG(19:1-18:0) | PG | PG(19:1-18:1) | PG | PG(19:1-18:1) |
| PG | PG(19:1-18:1) | PG | PG(19:1-19:1) | PG | PG(19:1-19:1) |
| PG | PG(19:1-19:1) | PG | PG(20:1-18:1) | PG | PG(20:1-18:1) |
| PG | PG(20:1-18:1) | PS | PS(16:0-19:2) | PS | PS(16:0-19:2) |
| PS | PS(16:0-19:2) | PS | PS(16:1-19:2) | PS | PS(16:1-19:2) |
| PS | PS(16:1-19:2) | PS | PS(17:1-19:2) | PS | PS(17:1-19:2) |
| PS | PS(17:1-19:2) | PS | PS(17:1-20:3) | PS | PS(17:1-20:3) |
| PS | PS(17:1-20:3) | PS | PS(17:1-21:3) | PS | PS(17:1-21:3) |
| PS | PS(17:1-21:3) | PS | PS(18:1-21:3) | PS | PS(18:1-21:3) |
| PS | PS(18:1-21:3) | PS | PS(19:2-14:0) | PS | PS(19:2-14:0) |
| PS | PS(19:2-14:0) | | | | |
